# Microtubule inhibitor, SP-6-27 inhibits angiogenesis and induces apoptosis in ovarian cancer cells

**DOI:** 10.18632/oncotarget.17549

**Published:** 2017-05-02

**Authors:** Arpita Kulshrestha, Gajendra K. Katara, Safaa A. Ibrahim, Renukadevi Patil, Shivaputra A. Patil, Kenneth D. Beaman

**Affiliations:** ^1^ Department of Microbiology and Immunology, Chicago Medical School, Rosalind Franklin University of Medicine and Science, North Chicago, USA; ^2^ Pharmaceutical Sciences Department, College of Pharmacy, Rosalind Franklin University of Medicine and Science, North Chicago, USA; ^3^ Department of Microbiology and Immunology, Faculty of Pharmacy, Cairo University, Giza, Egypt

**Keywords:** chromene, microtubule inhibitor, ovarian cancer, cisplatin resistance

## Abstract

In ovarian cancer (OVCA), treatment failure due to chemo-resistance is a serious challenge. It is therefore critical to identify new therapies that are effective against resistant tumors and have reduced side effects. We recently identified 4-H-chromenes as tubulin depolymerizing agents that bind to colchicine site of beta-tubulin. Here, we screened a chemical library of substituted 4-H-chromenes and identified SP-6-27 to exhibit most potent anti-proliferative activity towards a panel of human cisplatin sensitive and resistant OVCA cell lines with 50% inhibitory concentration (IC_50_; mean ± SD) ranging from 0.10 ± 0.01 to 0.84 ± 0.20 μM. SP-6-27 exhibited minimum cytotoxicity to normal ovarian epithelia. A pronounced decrease in microtubule density as well as G2/M cell cycle arrest was observed in SP-6-27 treated cisplatin sensitive/resistant OVCA cells. The molecular mechanism of SP-6-27 induced cell death revealed modulation in cell-cycle regulation by upregulation of growth arrest and DNA damage inducible alpha transcripts (GADD45). An enhanced intrinsic apoptosis was observed in OVCA cells through upregulation of Bax, Apaf-1, caspase-6, -9, and caspase-3. *In vitro* wound healing assay revealed reduced OVCA cell migration upon SP-6-27 treatment. Additionally, SP-6-27 and cisplatin combinatorial treatment showed enhanced cytotoxicity in chemo-sensitive/resistant OVCA cells. Besides effect on cancer cells, SP-6-27 further restrained angiogenesis by inhibiting capillary tube formation by human umbilical vein endothelial cells (HUVEC). Together, these findings show that the chromene analog SP-6-27 is a novel chemotherapeutic agent that offers important advantages for the treatment of ovarian cancer.

## INTRODUCTION

Ovarian cancer (OVCA) ranks fifth in the most prevalent cancer types in women with about 22,280 women diagnosed annually in the United States [1–4]. The standard OVCA treatment involving platinum-based chemotherapy often leads to the emergence of an unmanageable drug resistant phenotype [5–6]. The recent drugs designed to act against individual molecular targets are not effective in combating this multigenic disease [7]. Therefore, there is an urgent need for new alternate drugs with more efficient therapeutic impact on OVCA, especially in treating chemo-resistant phenotypes.

Microtubules are highly dynamic cytoskeletal structures consisting of α- and β- chains of tubulin [8]. Microtubules regulate critical cellular functions such as cell motility, intracellular trafficking and most importantly, cell growth and division. Targeted perturbation of microtubular dynamics represents the major therapeutic strategy to treat many types of cancers [9–11]. However, microtubular inhibitors anti-cancer agents pose limitations of toxic side effects and emergence of multi-drug resistant phenotypes [12]. Therefore, it is imperative to employ novel drug development approaches to design more effective microtubule targeting agents with fewer side effects [9–10, 13].

Based on their effect on tubulin assembly, microtubule disrupting agents can act as either microtubule stabilizing or destabilizing agents. Currently, in OVCA clinical therapeutics, microtubule stabilizing agents that target the taxane site of tubulin such as paclitaxel are widely used [14]. Paclitaxel has limitations such as transporter mediated drug resistance [15–16]. Drug discovery has now shifted the focus to microtubule destabilizing agents. This includes agents targeting the colchicine binding site. Currently, there are no approved drugs for colchicine site binding agents [17].

Chromenes are synthetically feasible analogs of the natural anti-mitotic compound lignin podophyllotoxin (PT) [18–20]. We recently identified 4H-chromenes as potent anti-cancer agent that binds to the colchicine site and causes tubulin de-polymerization [21–22]. We identified GRI-394837 and GRI-102696 as initial lead compounds. Based on GRI-394837, we designed, synthesized and screened a focused set of 4H-chromene against different cancer cell lines (glioma, melanoma and prostate) *in vitro*. The SP-6-27 emerged as the initial lead from our studies [23]. Here, we evaluated the efficacy of SP-6-27 towards ovarian cancer cells. Our findings demonstrate that SP-6-27 has potent anti-tumor activity towards both cisplatin sensitive and resistant ovarian cancer cells. The compound exhibited minimal cytotoxicity towards normal ovarian epithelial cells. SP-6-27 treatment led to the disruption of tubulin dynamics and induction of G2-M cell cycle arrest. Cell death pathway analysis revealed an induction of intrinsic apoptosis in caspase dependent manner (caspase-9 and -3). Additionally, SP-6-27 exerted inhibitory effects on the cancer cell migration. Further, the combination treatment of SP-6-27 and cisplatin exhibited enhanced cytotoxicity towards OVCA cells. In addition to the anti-cancer effects, SP-6-27 also displayed potent anti-angiogenic effects on human endothelial cells. These observations provide evidence that SP-6-27 is a novel microtubule disrupting agent effective against chemo-resistant ovarian cancer cells as well as disrupts angiogenesis of endothelial cells.

## RESULTS

### Microtubule inhibitor SP-6-27 shows cytotoxicity towards a panel of ovarian cancer cell lines

**Figure 1:**
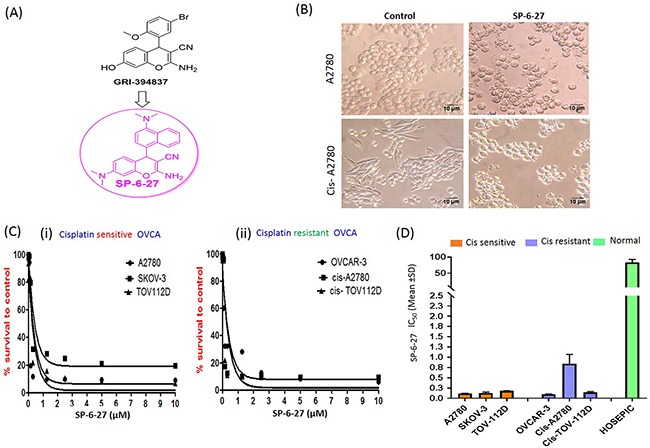
Microtubule inhibitor SP-6-27 exhibits cytotoxicity towards human ovarian cancer cells **(A)** Structure of SP-6-27 as derived from the parent compound GRI-394837. **(B)** Changes in the cell morphology of the ovarian cancer cells upon treatment with SP-6-27. Cisplatin sensitive (A2780) and resistant (cis-A2780) ovarian carcinoma cells were incubated with vehicle control (DMSO) or SP-6-27 (0.5 μM, 24 h). Cell morphology was assessed by phase-contrast microscopy and representative images (20X magnification; scale bar- 10 μm) are shown here. **(C)** Dose–response curves of SP-6-27 in a panel of **(i)** cisplatin sensitive (A2780, SKOV-3, TOV-112D) and **(ii)** cisplatin resistant (OVCAR-3, cis-A2780, cis-TOV-112D) ovarian cancer cell lines following 72h treatment. **(D)** IC_50_ values (mean± SD) for SP-6-27 in different human ovarian cancer cell lines compared to normal ovarian epithelial cells (HOSEpiC). The cell viability was assessed by Alamar Blue assay. Data represents mean of 3 independent experiments performed at least in triplicate.

First, we investigated the effects of 4-H chromene analogs on the ovarian cancer cell viability. Out of the several 4-H-chromene derivatives, SP-6-27 exhibited potent cytotoxicity in A2780 ovarian cancer cells with IC_50_ values below 10 μM [Supplementary Figure 1]. We selected SP-6-27 to study its effect on the viability of a panel of six human ovarian cancer cell lines. The structure of the SP-6-27 is shown in [Figure 1 (A)]. Upon treatment with SP-6-27 (0.5 μM, 24h), the adherent ovarian carcinoma cells showed a change in morphology with more rounded cells along with cell shrinkage [Figure 1 (B)]. The SP-6-27 dose response curves for the cisplatin sensitive cell lines A2780, SKOV-3 and TOV-11D suggested growth inhibition [Figure 1 (C i)] with the IC_50_ (mean ± SD) of 0.14± 0.03 μM. SP-6-27 exhibited similar cytotoxicity against cisplatin resistant cell lines OVCAR-3, cis-A2780 and cis-TOV-112D with mean IC_50_ values of 0.36± 0.41 μM [Figure 1 (C ii)]. Overall, the IC_50_ (mean ± SD) ranged from 0.10 ± 0.006 – 0.84±0.23 μM after 72 h of incubation for all the OVCA cell lines tested [Figure 1 (D)]. The usefulness of an anticancer agent also depends on the absence of toxicity toward normal cells. We therefore evaluated the effect of SP-6-27 on the normal ovarian epithelial cells (HOSEpiC). No cytotoxic effect was observed towards HOSEpiC cells at the same SP-6-27 concentrations that were lethal for OVCA cells (IC_50_ mean ± SD: 83.35 ± 9.47 μM) [Figure 1 (D)].

### SP-6-27 causes disruption of microtubular dynamics in ovarian cancer cells

**Figure 2:**
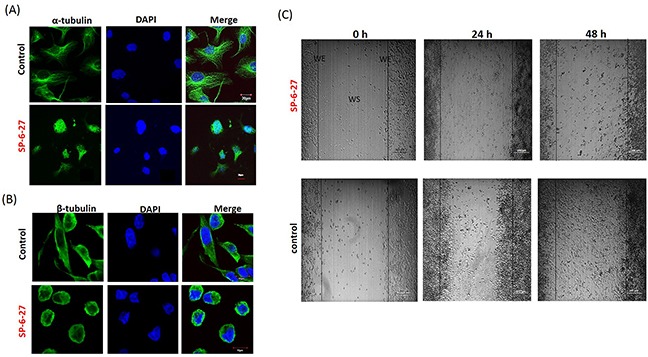
Chromene analog SP-6-27 disrupts the microtubular dynamics and inhibits migration in ovarian cancer cells Ovarian cancer cells (A2780) were treated with control (DMSO, vehicle) or SP-6-27 (0.5μM) for 24 h and stained with DAPI (blue) and an **(A)** anti–α-tubulin or **(B)** anti-β-tubulin antibody (green). Images were captured using an Olympus FluoView confocal microscope. Representative confocal micrographs (original magnification: 80X) are shown. **(C)** Effect of SP-6-27 on OVCA cell migration. *In vitro* wound healing assay was performed using A2780 cells cultured in 6 well plates. Confluent cultures were scratched with a 1 mL pipette tip as described in the Methods section. Representative phase-contrast images of cells migrating into the wounded area in SP-6-27 treated and control wells (0, 24 and 48 h) are shown here. W: wound space, WE: wound edge (magnification- 4X, scale bar-200 μm).

In order to understand the mechanism of action of SP-6-27 that affects the OVCA cell survival, we investigated the microtubular dynamics in SP-6-27 treated cells. The microtubule assembly at the cellular level was studied using immune-fluorescence analysis. OVCA cells were exposed to SP-6-27 (0.5 μM; 24 h) and the fixed cells were stained with anti- α- or β-tubulin antibodies. Compared to vehicle control, SP-6-27 treated cells demonstrated an aberrant cytoskeletal destruction with disruption of α-tubulin [Figure 2 (A) and Supplementary Figure 2] and β tubulin [Figure 2 (B)].

Tumor cell migration is a critical step in tumor growth/metastasis and microtubules are imperative to this process [24–25]. The effect of SP-6-27 on tumor cell migration was evaluated using monolayer wound healing assay. Monitoring the cell movement over 48 h showed that the migration was reduced in A2780 cells upon treatment with SP-6-27 (0.5 μM) compared to the control cells [Figure 2 (C)].

### SP-6-27 causes G2-M cell cycle arrest in ovarian cancer cells

**Figure 3:**
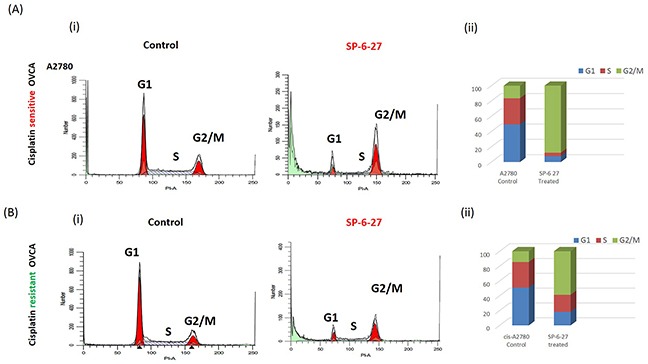
Cisplatin sensitive and resistant ovarian cancer cells arrest in G2 and M phase following SP-6-27 treatment Cisplatin sensitive A2780 or cisplatin resistant cis-A2780 ovarian cancer cells were treated with 0.5μM SP-6-27 or DMSO vehicle control for 24 hours. The cells were evaluated for effects on cell cycle using PI staining and analyzed by flow cytometry using ModFit software. **(Ai)** Representative cell cycle micrographs of cisplatin sensitive cells depicting G1, S and G2/M cell populations in control and SP-6-27 treated cells. **(Aii)** Stacked bar graph illustrating the phase distribution of cisplatin sensitive cells in control and SP-6-27 treated groups determined as percentage of the total number of cells in cycle. **(Bi)** Representative cell cycle micrographs of cisplatin resistant cells depicting G1, S and G2/M cell populations in control and SP-6-27 treated cells. **(Bii)** Stacked bar graph illustrating the phase distribution of cisplatin resistant cells in control and SP-6-27 treated groups. The data indicates ovarian cancer cell cycle arrest in G2/M phase upon SP-6-27 treatment. Average of three experiments is shown.

Microtubule dynamics plays an important role in cell cycle progression and its disruption may either lead to mitotic arrest or mitotic exit, ultimately leading to cell death [26–27]. To determine if the SP-6-27 mediated ovarian cancer cell growth and migration inhibition is due to cell-cycle perturbation, we studied the distribution of cells in different phases of the cell cycle by Flow cytometry. The OVCA cells are primarily in G1 and S phase. SP-6-27 treatment caused a complete collapse of all the cells in G1 phase. There was a substantial increase in G2-M cells indicating mitotic arrest in G2-M [Figure 3 (A i and B i) and Supplementary Figure 3]. This G2-M cell cycle arrest was evident in both cisplatin sensitive and resistant cells. The cell cycle distribution of cisplatin sensitive and resistant OVCA cells after SP-6-27 treatment is shown in Figure 3 (A ii and B ii). In the A2780 cell line, 87.8 ± 6.2% cells were arrested in G2-M phase compared to 16.40 ± 6.2% cells in the control group. In the cisplatin resistant cis-A2780 cell line, 58.9 ± 3.4% cells were arrested in the G2/M phase compared to 14.5 ± 0.2 % cells in the control group. This is in line with studies indicating that the tubulin targeting drugs elicit a mitotic arrest in cancer cells [28–29]. Collectively, the data indicates a significant increase in the G2/M cell population.

### SP-6-27 induces apoptosis in ovarian cancer cells

**Figure 4:**
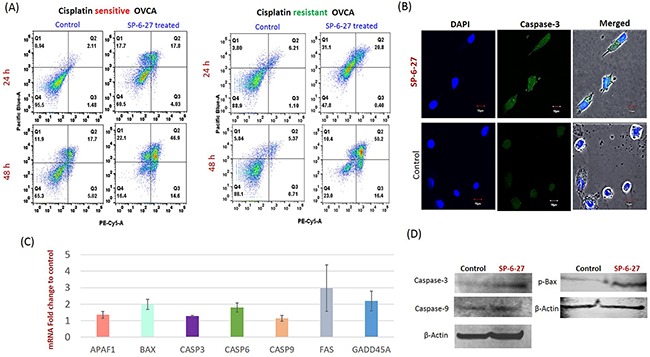
SP-6-27 induces apoptotic cell death in chemo-resistant and sensitive ovarian cancer cells **(A)** Cisplatin sensitive (A2780) and cisplatin resistant (cis-A2780) ovarian cancer cells were treated with 0.5 μM SP-6-27 (24, 48 h) followed by Annexin V/7-AAD assay. Early apoptotic [first (Q1) quadrant], late apoptotic [second (Q2) quadrant] and dead cells [(Q3) quadrant] are shown. **(B)** Confocal microscopy analysis for cleaved caspase-3 in SP-6-27 treated (0.5μM, 24h) ovarian cancer cells. Images show active caspase-3 expression in **(i)** SP-6-27 treated cells compared to **(ii)** control cells. Nuclei staining was performed using DAPI (blue). Merged phase-contrast-fluorescence microscopy images displaying cellular expression of active Caspase-3 and DAPI are shown here. Original magnification-× 80; Scale bars-10μm. **(C)** Q-RT-PCR array was performed for identification of the cell death pathway associated genes altered in ovarian cancer cells upon SP-6-27 treatment. mean ±SD of the mRNA fold change using two endogenous controls (BACT and HPRT) is depicted here. Data are the average of triplicate experiments [mean ± (SD)]. **(D)** Western blot analysis of the intrinsic apoptotic molecules in SP-6-27 treated A2780 ovarian cancer cells show enhanced expression compared to control cells.

The changes in OVCA cell viability can be a result of altered proliferation or an altered apoptosis and cell viability assays do not distinguish between the two. For this, the apoptosis was measured by Annexin-V assay using flow cytometry. As shown in Figure 4 (A), SP-6-27 treatment caused a pronounced increase in the Annexin-V positive cell population in cisplatin sensitive and resistant OVCA cells. Caspase-3 is the “executioner” caspase that is involved in the final irreversible step of the apoptotic pathway. We evaluated the presence of cleaved- active caspase-3 using immunofluorescence analysis. A2780 OVCA cells incubated with 0.5 μM SP-6-27 for 24 h showed an increase in caspase-3 activity compared with control cells [Figure 4 (B) and Supplementary Figure 4]. This suggests that SP-6-27 effectively induces apoptosis in ovarian cancer cells.

### SP-6-27 modulates pro-apoptotic molecules leading to enhanced cell death

Expression profiling of the cell death pathway was performed to determine which genes are modulated by SP-6-27 that contribute to cytotoxicity towards OVCA cells. We found a significant upregulation (p<0.05) of the pro-apoptotic genes including Bax, APAF-1, caspase-9, caspase-6, Caspase-3, indicating the involvement of intrinsic apoptotic pathway [Figure 4 (C)]. The upregulation of Fas gene was also observed. The qPCR array data for the selected apoptotic genes was validated by individual qPCR in 4 ovarian cancer cell lines (Supplementary Figure 5). The intrinsic apoptotic pathway induces Bax/Bak activation and subsequent permeabilization of the mitochondrial membrane, leading to the activation of caspases. Further, upregulation of intrinsic apoptotic molecules (caspase-9, p-Bax and caspase-3) was further confirmed at protein level by western blot analysis [Figure 4 (D)].

In addition to apoptosis associated genes, we found an upregulated expression of GADD45A (p<0.05) which plays an important role in cell cycle G2-M arrest in response to genotoxic stress [Figure 4 (C)].

### Combination of SP-6-27 and cisplatin shows enhanced cytotoxicity on cisplatin sensitive and resistant cells

**Figure 5:**
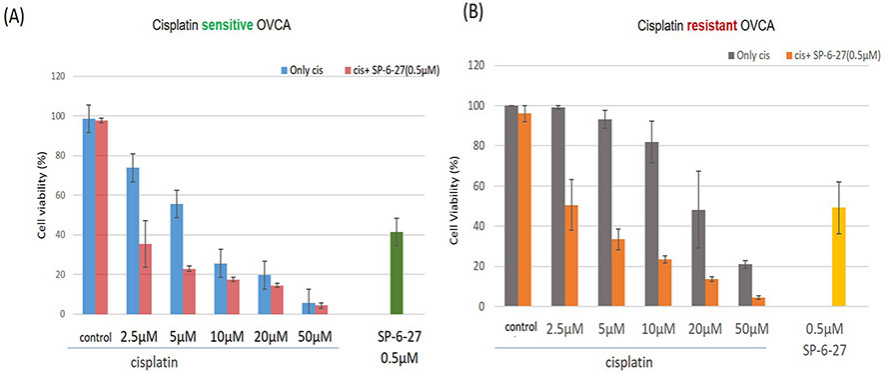
SP-6-27 and cisplatin combination treatment exhibits enhanced cytotoxicity towards ovarian cancer cells **(A)** The cell viability of cisplatin sensitive cells (A2780) following simultaneous treatment with a combination of SP-6-27 and cisplatin. Blue columns, cells treated with cisplatin only (0-50μM, 48h); red columns, cells treated with combination of cisplatin (0-50μM) and 0.5 μg SP-6-27 for 48h; green column shows cells treated with SP-6-27 alone (0.5 μM, 48h). **(B)** Sequential combinatorial treatment with SP-6-27 (0.5μM; 24h) followed by cisplatin (0- 50μM; 24h) enhanced cytotoxicity in the of cisplatin resistant cells (cis-A2780) cells. Gray columns, cells treated with cisplatin only; orange column, cells treated with a combination of cisplatin and SP-6-27; yellow column shows cells treated with SP-6-27 (0.5 μM, 48h) alone. The experiments were performed twice in triplicate.

To investigate the utility of SP-6-27 in combinatorial anticancer treatment, we simultaneously treated OVCA cells with SP-6-27(0.5 μM) and cisplatin (2.5, 5, 10, 20 and 50 μM) for 48h. Combination treatment of SP-6-27 with cisplatin resulted in significant cell growth inhibition in ovarian cancer cells. This simultaneous combination treatment was effective in in killing the cisplatin sensitive cells [Figure 5 (A)]. On the contrary, in cisplatin resistant cells, the concomitant addition of cisplatin blocked SP-6-27-induced cell death (data not shown). This is in line with the previous observations where it was found that cisplatin can inhibit the effectiveness of paclitaxel in cis-resistant cell lines [30]. As an alternate strategy, we employed sequential treatment in cisplatin resistant cells. Pre-treatment with SP-6-27 (0.5 μM, 24h) followed by cisplatin (2.5-50 μM, 24 h) showed an enhanced cytotoxicity compared to concurrent treatment approach [Figure 5 (B)]. This suggests that to be effective in cisplatin resistant cells, precise sequencing is particularly important for combinatorial treatment with SP-6-27 and cisplatin.

### SP-6-27 inhibits tube formation in vascular endothelial cells

**Figure 6:**
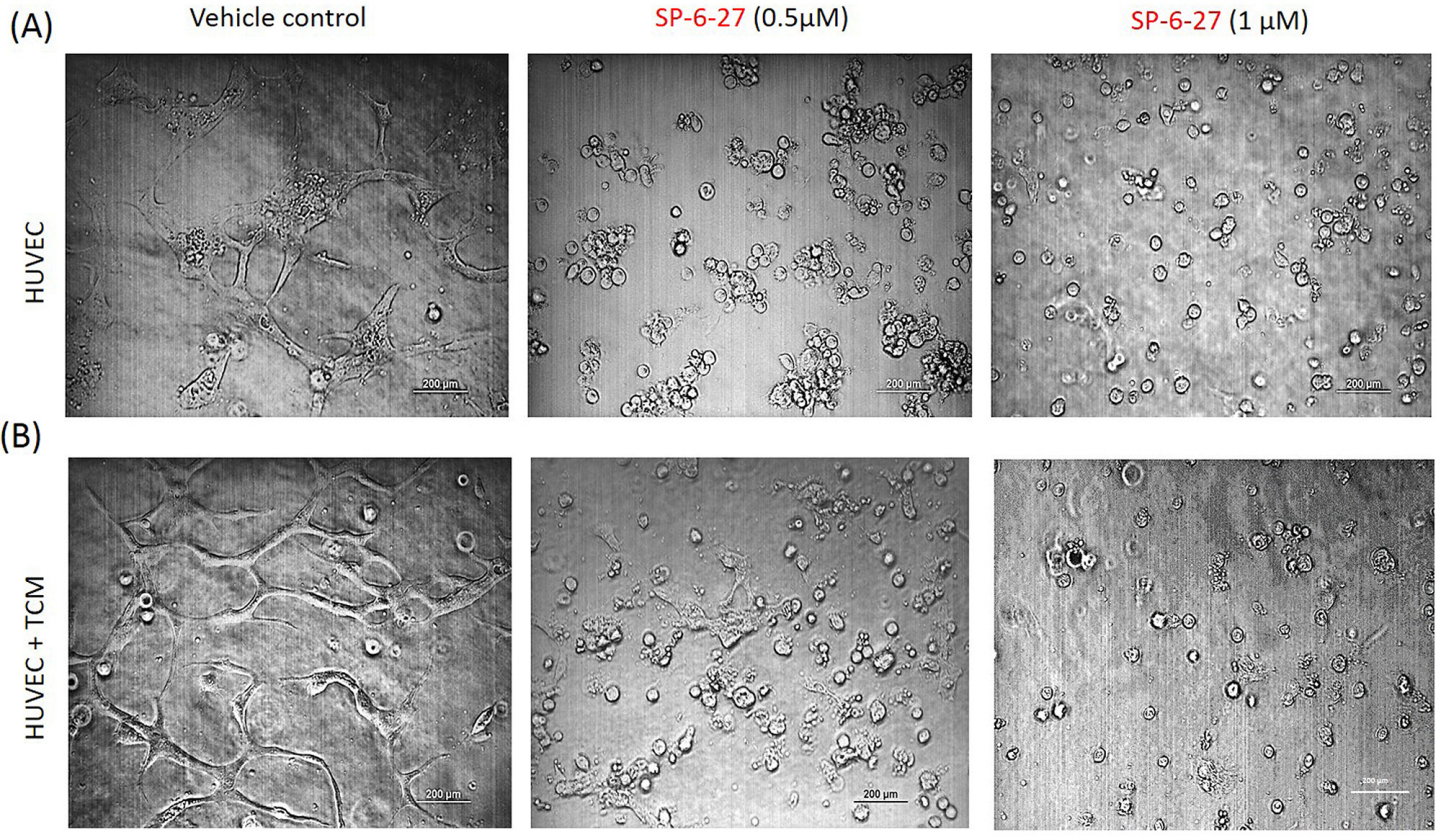
SP-6-27 inhibits capillary tube formation by endothelial cells HUVECs endothelial cells were grown on matrigel-coated 96-well plates for 24 h. The cells were treated with SP-6-27 (0.5, 1μM) or DMSO vehicle, in the **(A)** absence/**(B)** presence of tumor conditioned media from ovarian cells. The ability of the HUVEC cells to form capillary tube like structures was analyzed using phase contrast microscopy at a magnification of ×4, scale bars- 200μm. Images shown are representative of three independent experiments.

Endothelial cells are important in angiogenesis and their proliferation is critical in tumor metastasis [31]. Previously, colchicine site microtubule inhibitors have shown their efficacy in inhibiting angiogenesis by acting as vascular disrupting agents that disrupt the endothelial vasculature [32]. To elucidate the possible role of SP-6-27 as a vascular disrupting agent, its effect on the tube formation potential of endothelial cells was assessed *in vitro*. Angiogenesis can be estimated by the ability of the endothelial cells to form 3 dimensional capillary tubes in the matrix. At 24h, SP-6-27 strongly inhibited the endothelial cell tube formation at 0.5μM or 1 μM concentration compared to control cells [Figure 6 (A)]. Further, SP-6-27 completely prevented capillary formation in the presence of ovarian tumor conditioned media [Figure 6 (B)]. The results strongly suggest the efficacy of SP-6-27 in vascular disruption leading to suppressed angiogenesis.

## DISCUSSION

Ovarian cancer is the leading cause of death among gynecological malignancies, the primary reason being the frequent disease relapse, mostly with a drug resistant phenotype [33]. Novel therapies effective towards chemo-resistant profile are therefore required to improve the survival rates. Microtubule dynamics plays critical role in diverse biological processes such as cell division, cellular shape and motility. The microtubule-targeting agents have therefore been an important mainstay in anti-cancer therapy over the past several decades [19]. However, the clinical efficacy of the current microtubule targeting agents such as taxane binding drugs (paclitaxel and docetaxel) towards ovarian cancer is limited due to drug resistance and toxicity [34]. Moreover, microtubule destabilizing agents targeting the vinca site (vincristine, vinblastine) are as not effective in ovarian cancer as the platinum and taxanes and exhibit significant neurological toxicities [35–36]. Recent exploratory studies in patients have looked at treating at treating ovarian cancer patients with a novel vinca-alkaloid which was targeted to ovarian cancer cells specifically via folate receptor, which is over-expressed in many ovarian cancer cells. However, it did not prove very effective [37]. An alternate microtubule targeting site, the colchicine-binding site has attracted much attention in anti-cancer drug discovery. Several colchicine- binding agents, such as combretastatins are in phase trials for their anticancer activity [38]. However, there are currently no FDA approved drug of this class on the market.

**Figure 7:**
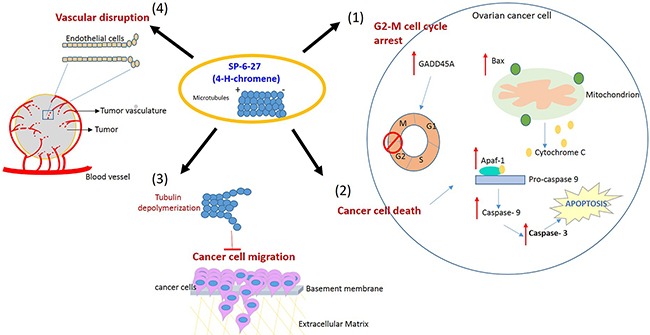
Proposed model illustrating the effects of SP-6-27 mediated microtubule inhibition on ovarian cancer cells and endothelial cells Schematic of the pleotropic effects of SP-6-27 on ovarian cancer growth and angiogenesis. Tubulin de-polymerization caused by SP-6-27 leads to (1) G2-M phase cell cycle arrest in these cells. (2) Enhanced apoptotic cell death- The elevated expression of Bax contributes to the mitochondrial cytochrome c mediated activation of caspase -9 involving Apaf-1. The activated caspases-9 further cleaves procaspase-3 and subsequently leads to apoptosis of the cell. (3) Reduced cancer cell migration- SP-6-27 mediated tubulin de-polymerization suppresses ovarian cancer cell migration. (4) Vascular disruption- SP-6-26 causes vascular disruption of the endothelial cells, thus inhibiting angiogenesis. The endothelial vascular disruption is evident in the presence of ovarian cancer tumor conditioned medium.

4*H*-Chromene derivatives are an important scaffold representing a class of naturally occurring benzopyran derivatives and their biological activity has been established as anti-microbial, anticancer, and as potent apoptosis inducers [39]. The current study highlights the utility of SP-6-27, a substituted 4-H chromene as a novel colchicine site targeting agent in cisplatin resistant ovarian cancer. The mechanism of cell death involved the disruption of microtubules, cell cycle arrest in G2/M phase that ultimately leads to caspase dependent apoptosis. This is in line with the previous studies where colchicine site inhibitors were shown to induce cell cycle arrest at the G2/M stage of the cell cycle, and promote cell death either by apoptosis or mitotic catastrophe [40–41]. Additionally, SP-6-27 treatment suppressed ovarian cancer cell migration. Microtubule inhibitors have been shown to interfere with cell migration by eliciting microtubule dynamic instability [42]. The combinatorial treatment of SP-6-27 with cisplatin showed enhanced anti-cancer effects on OVCA cells. Further, as an indicator of repressed angiogenesis, the SP-6-27 disrupted the tube formation ability of endothelial cells. This study establishes SP-6-27 as a novel class of chemotherapeutic agent that offers advantages for the treatment of chemo-resistant ovarian cancer [Figure 7].

The anti-proliferative activity of SP-6-27 revealed a cytotoxic effect on ovarian cancer cells with comparable efficacies towards cisplatin sensitive or resistant cells. This indicates that the mechanism of cell death induction by SP-6-27 is not compromised by anti-apoptotic mechanisms in cisplatin resistant cells. The chromene compounds exhibit anti-cancer activity by targeting microtubular dynamics [19]. The mechanism of cell death by SP-6-27 involved an enhanced apoptotic pathway. Previous studies have highlighted that the microtubule inhibitors modulate the cellular biological processes and signaling pathways, leading to apoptosis [43–44].

We have previously shown that SP-6-27 inhibited tubulin polymerization in a concentration dependent manner [22, 23]. Here, we further validated that the microtubules are the direct targets of SP-6-27. Microtubule binding by SP-6-27 caused complete destruction of the intricate microtubule network and the bundling of α and β tubulins, indicating a decrease in polymerized tubulin. Microtubule destabilizing agents are known to cause tubulin de-polymerization leading to cytoskeletal destruction that leads to apoptosis [29].

SP-6-27 mediated inhibition of tubulin poly-merization led to a complete blockade of the cell cycle at the G2/M phase in cisplatin sensitive and resistant OVCA cells. This is in corroboration with previous reports highlighting that the microtubule-binding agents arrest the cell cycle at G2/M phase ultimately leading to cell death [29]. ‘Mitotic catastrophe’ is defined as cell death during mitosis due to DNA damage or the debilitated checkpoint mechanisms that are involved in arrest and repair of cells. Mitotic catastrophe can be induced by microtubule targeting agents (including the tubulin stabilizing and de-stabilizers) [45]. G2-M cell cycle progression requires the activation of the Cdk1/cyclin B1 complex. Due to the deficiency of cell-cycle checkpoints in cancer cells, mitotic catastrophe can be easily induced in these cells using microtubule inhibitors. Further, the cdk1 expression can be modulated in p53 dependent manner [46] and can also be enhanced by increased transcription of Cdk1 inhibitors that include Gadd45, p21 and 14-3-3s [47]. In the present study, SP-6-27 treatment enhanced expression of GADD45 transcription in OVCA cells that may be responsible for the inhibition of cyclinB1/cdk1 complex, resulting in G2-M cell cycle arrest.

The induction of apoptotic cell death was confirmed by Annexin-V apoptosis assay. Apoptotic cell death induction is regulated by caspase proteins, which are present as pro-enzymes. The activation of caspases in turn results in the cleavage and inactivation of many cellular proteins leading to apoptotic cell death in many cell types [48]. Caspases have been divided into initiator caspases (caspases-2, -8, -9, and -10) and effector caspases (caspases-3, -6, and -7) [49]. In addition to intrinsic pathway molecules, Fas and TNF-R were also upregulated. Microtubule stabilizing agents such as taxanes prevent cell cycle completion and thus lead to apoptotic cell death [50]. Microtubule de-polymerizing agents such as vinblastine and vincristine also exhibit similar effect. In contrast to DNA damaging agents, apoptosis triggered by disruption of microtubule dynamics is associated with bcl-2 phosphorylation [51]. In the present study, the results show that SP-6-27 induced activation of caspase-3, which is the key effector caspase indicating the involvement of caspase mediated apoptotic pathway in SP-6-27 induced cell death. The cell death array revealed upregulation of Bax, Apaf-1 and caspase 9 indicating the involvement of intrinsic apoptotic pathway.

The targets of microtubule affect both cancer cells and vascular endothelial cells [50]. Endothelial cell based vasculature is required by tumor cells for growth and metastasis. Vascular disrupting therapy has therefore become an attractive strategy to disrupt the established tumor vasculature by directly effecting the endothelial cells and indirectly cause necrosis of the tumor mass [52–54]. Furthermore, vascular disruption is an effective anti-cancer strategy irrespective of the chemo-sensitive/resistant tumor cell phenotype. Several microtubule depolymerizing agents have shown potent vascular disrupting properties due to their capacity to target the cytoskeleton and compromise the integrity of endothelial cell junctions [55–56]. Recently, the combination of vascular disrupting agent combretastatin A4 phosphate (CA4P; fosbretabulin) with carboplatin and paclitaxel was found effective in patients with platinum-resistant ovarian cancer [57]. In line with these studies, SP-6-27 potently blocked the capillary-like tube formation by endothelial cells. This inhibition was observed even in the presence of tumor cell conditioned media (contains potent angiogenic factors), indicating the efficacy of SP-6-27 treatment in established tumor vasculature. Our results therefore provide the evidence that SP-6-27 exerts a potent vascular disrupting effect. Previous studies have suggested the utility of microtubule-binding agents towards vascular-targeted therapy [18].

In summary, the development of effective and safe anti-cancer agents is an essential step to overcome treatment failure towards ovarian cancer therapy. We identified the dual role of colchicine site inhibitor SP-6-27 as an anti-cancer as well as vascular disrupting agent in ovarian cancer, making it a promising potential antitumor drug candidate for further development.

## MATERIALS AND METHODS

### Library screening for chromene analogs

#### SP-6-27 stocks

Chromene analogs, SP-6-27 were synthesized as described previously [22]. SP-6-27 compound was a solid powder and was reconstituted with DMSO to yield concentrations of 50 mM. Stocks were stored as 20 μL aliquots at −80°C protected from light.

### Cell culture

Six human OVCA adenocarcinoma cell lines PA-1, CaOV-3, OVCAR-3, A2780, TOV112D and SKOV-3 were employed in the study. Out of these, OVCAR-3 cell line has its origin from cisplatin refractory patient. Additionally, two acquired cisplatin resistant OVCA cell lines, cis-A2780 and cis-TOV-112D were also employed to assess their sensitivity towards chromene analogs. The culture conditions for these cell lines have been described previously [58]. All media were supplemented with 10% FBS, 1% L-glutamine, and 1% antibiotic solution of penicillin/streptomycin. Normal human ovarian epithelial cells (HOSEpic; ScienCell, Carlsbad, CA, USA) were employed as control cells. HOSEpic cells were grown in HOSEpic cultivation medium (ScienCell, USA) in poly-L lysine coated flasks. All cells were cultivated at 37°C in a humidified atmosphere containing 5% (v/v) CO_2_. For routine culture, cells were grown until reaching approximately 80% confluence followed by sub cultured or plating for experiments.

### Cell viability assays

Cells were seeded in 96-well plates (0.25 × 10^4^ cells/well) and allowed to attach overnight. The plated cells were exposed to different drug concentrations (0.001−10 μM). For determining the cell viability at the 72 hr time point, the drug containing medium was removed and cells were washed twice with PBS. The Alamar Blue reagent (Life Technologies, Grand Island, NY) was added for 1-2 h, 37°C, 5% CO_2_. The fluorescence reading was taken at fluorescence excitation wavelength of 540 nm and emission at 580 nm. The half-maximal inhibitory concentration (IC_50_) was determined using Origin6.0 software (Origin Labs, MA, USA) and dose response curves were generated using GraphPad Prism 5.0 software (GraphPad, San Diego, CA, USA).

### Confocal microscopy for tubulin cytoskeletal structure

OVCA cell lines were plated in 8-well chamber slides (Nunc, USA) at 3000 cells/well and were incubated overnight in 5% CO_2_ at 37°C. Cells were subsequently treated with SP-6-27 (0.5 μM) or DMSO vehicle control. After 24 h, cells were washed with PBS, fixed with 4% paraformaldehyde for 25 min at room temperature (RT) and permeabilized with methanol: acetone (9:1) for 5 min at −20°C. Slides were blocked with image-iT FX Signal Enhancer (Thermo Fisher Scientific) for 45 min at RT. Primary antibody staining for alpha-tubulin (1:250), beta tubulin (1:250) and caspase-3 (1:250) [Abcam, MA, USA] was performed by incubating the slides for 2 h at RT. The cells were incubated with secondary antibody Alexa Fluor^®^ 488-conjugated goat donkey-anti Rabbit (Invitrogen, USA) for 45 min at RT. The cells were prepared for viewing using SlowFade® Gold Antifade Mountant with DAPI (Invitrogen). For confocal microscopy, the stained cells were imaged by Fluoview Fv10i confocal microscope (Olympus). Analysis was performed using Fv10i Flouview Ver.3.0 software. Experiments were repeated at least twice in triplicate.

### *In vitro* wound healing/migration assay

For wound healing assay, OVCA cells (A2780) were allowed to grow up to confluency in 24 well plates. Three separate wounds were scratched through the cells using a sterile 1 ml pipet tip. The wells were then gently washed with PBS and fresh media containing 0.5μM SP-6-27 or DMSO control was added. The wells were assessed at the beginning 0, 24 and 48h incubation. The images were captured using TE100 Nikon inverted microscope at 4X magnification. The assay was repeated thrice in duplicate.

### Apoptosis assay

OVCA cells were seeded in 6 well plates (TPP Techno Plastic Products) at a density of 0.25 X10^5^ cells/well and allowed to attach overnight. Different OVCA cells were exposed to 0.5 uM SP-6-27 concentration or with DMSO vehicle control. After 24 or 48 hrs, floating and attached cells were collected, and washed with PBS. Annexin V /7 AAD staining (BD Pharmingen, San Diego, CA, USA) was used to detect apoptosis according to the manufacturer’s instructions. For this, 1 × 10^5^ cells were resuspended in 250 μl of binding buffer and 5 μl of Annexin V-fluorescein isothiocyanate and 5 μl of 7-AAD. Cells were incubated at room temperature for 15 min in dark, after which the cells were washed, resuspended in 300 μl binding buffer and immediately analyzed in flow cytometer (FACSCalibur, Becton Dickinson, Franklin Lakes, NJ, USA). The experiment was performed at least twice in triplicate

### Cell-cycle analysis

In order to detect the distribution of cells within the different phases of cell cycle, cells were stained with PI using BD Cycletest™ Plus DNA kit (BD Biosciences, USA) and analyzed by flow cytometry. Briefly, 0.5 × 10^6^ cells of control or SP-6-27 treated (0.5 uM, 24h) cells were trypsinized and washed with PBS. Cells were treated with a series of buffers for digestion of cell membranes, cytoskeletons and RNA according to the manufacturer instructions. PI staining was performed for 10 min at 4°C. Immediately, the fluorescence emitted from the PI–DNA was measured for individual cells using a flow cytometer (BD, FACS Calibur). The data was acquired using BD CellFIT software. Data from three experiments (in triplicate) were collected and mean ± S.D. of the percentage of cells in the G_2_/M phase were calculated.

### RNA isolation and reverse transcription-PCR

RNA isolation from cultured cells was performed using RNeasy® mini kit (Qiagen, USA) according to the manufacturer’s protocol. RNA Samples were stored at −80°C until further use. For cDNA preparation, 1μg of total RNA was reverse transcribed at 37°C using high capacity cDNA kit according to manufacturer`s instructions (Applied Biosystems, Foster City, CA). For cell death pathway analysis (RT2 profiler, SA Biosciences, Frederick, MD, USA), PCR array based expression profiling was performed using SYBR-Green method and the results were analyzed using the ΔΔCt method using RT^2^ profiler PCR data Analysis software version 3.5 (SA Biosciences).

### Combinatorial treatment studies

The ovarian tumor cells were treated with either the simultaneous or sequential combination of cisplatin and SP-6-27. For simultaneous combination assays, 10,000 cells were plated in each well of 96 well plate. After O/N incubation, cells were treated with (i) cisplatin alone at 2.5, 5, 10, 20 and 50 μM concentration, (ii) SP-6-27 alone at 0.5μM, or (iii) different concentrations of cisplatin (2.5, 5, 10, 20, 50 μM) plus SP-6-27 (0.5 μM) for 48 h at 37°C, 5% CO_2_. For sequential combination assays, 10,000 cells were plated in each well of 96 well plate. After O/N incubation, cells were treated with SP-6-27 at 0.5μM or treated with DMSO vehicle control for 24h. Medium was removed and cells were treated different concentrations of cisplatin (2.5, 5, 10, 20, 50 μM) for 24 h at 37°C, 5% CO_2_. After 48 h, percent cell viability compared to single drug treatment was determined using colorimetric MTS assay (Promega, USA) as recommended by the manufacturer.

### Capillary-like tube formation/angiogenesis assay

The anti-angiogenesis ability of SP-6-27 *in vitro* as evaluated using capillary tube formation assay as described previously [59]. Briefly, 96-well plate was coated with Matrigel at 37°C for 1h. HUVEC endothelial cells (15,000 /well) were suspended in EGM medium and seeded onto the Matrigel coated wells. The cells were treated with vehicle control, SP-6-27 (0.5, 1μM), tumor conditioned media (positive control) and SP-6-27 in the presence of tumor conditioned media (all treatments in triplicate) for 24h. The tube formation was assessed with TE100 Nikon inverted microscope and images were captured at 4X magnification.

### Statistical analysis

All experiments were repeated at least thrice in triplicate, with representative experiments demonstrated. The means of the data sets were compared and significance was determined by two-tailed Students t-test. Differences were considered to be statistically significant where P<0.05. Data is graphically represented as the standard deviation of the mean (SD). Data was analyzed using GraphPad Prism (version 5) statistical software. Dose response curves were generated as semi-log plots where the drug dose had a wide range. Origin 6.0 software was used for determination of the IC50 values using sigmoidal regression analysis.

## SUPPLEMENTARY MATERIALS FIGURES


