# Supplementary material for: Microtubule inhibitor, SP-6-27 inhibits angiogenesis and induces apoptosis in ovarian cancer cells
Source: Oncotarget. 2017 May 2;8(40):67017–28. doi: 10.18632/oncotarget.17549 (PMC5620153; doi:10.18632/oncotarget.17549)
Supplement: Supplementary file 1 [file oncotarget-08-67017-s001.pdf]

Microtubule inhibitor, SP-6-27 inhibits angiogenesis and induces apoptosis in ovarian cancer cells

SUPPLEMENTARY MATERIALS

4-H chromene	Structure	IC_{50} (mean)
SP-6-Ch 1	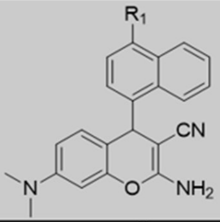	5.3 µM
SP-6-Ch 2	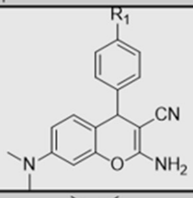	>10 µM
SP-6-27	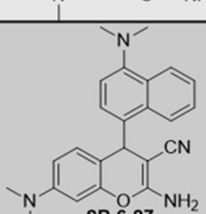 SP-6-27 $C_{24}H_{24}N_4O$ (384.47)	0.2µM
SP-6-Ch 3	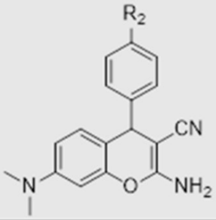	>10 µM
SP-6-Ch 4	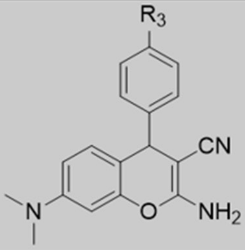	>10 µM

Supplementary Figure 1: IC_{50} of the tested chromene analogs as determined at 72 h exposure by Alamar Blue assay.

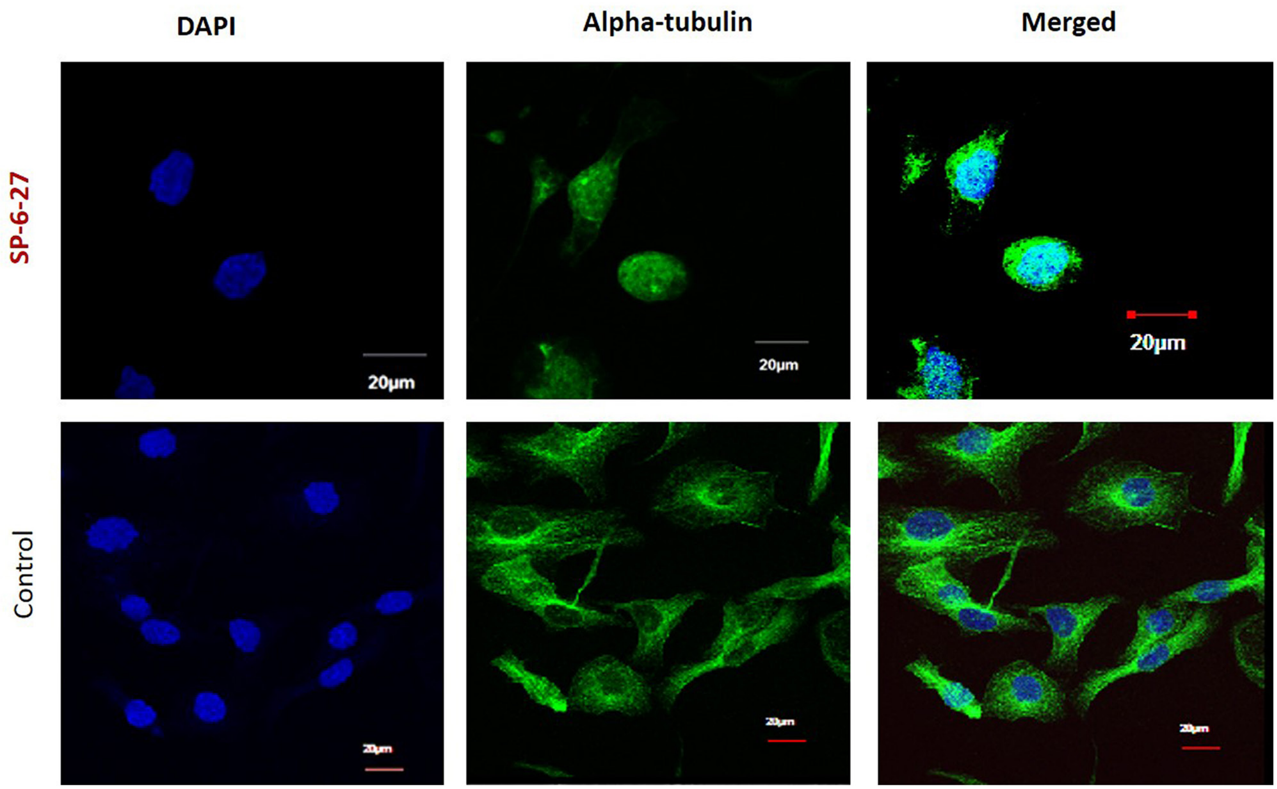

Supplementary Figure 2: Chromene analog SP-6-27 disrupts alpha-tubulin dynamics in ovarian cancer as determined by confocal microscopy analysis.

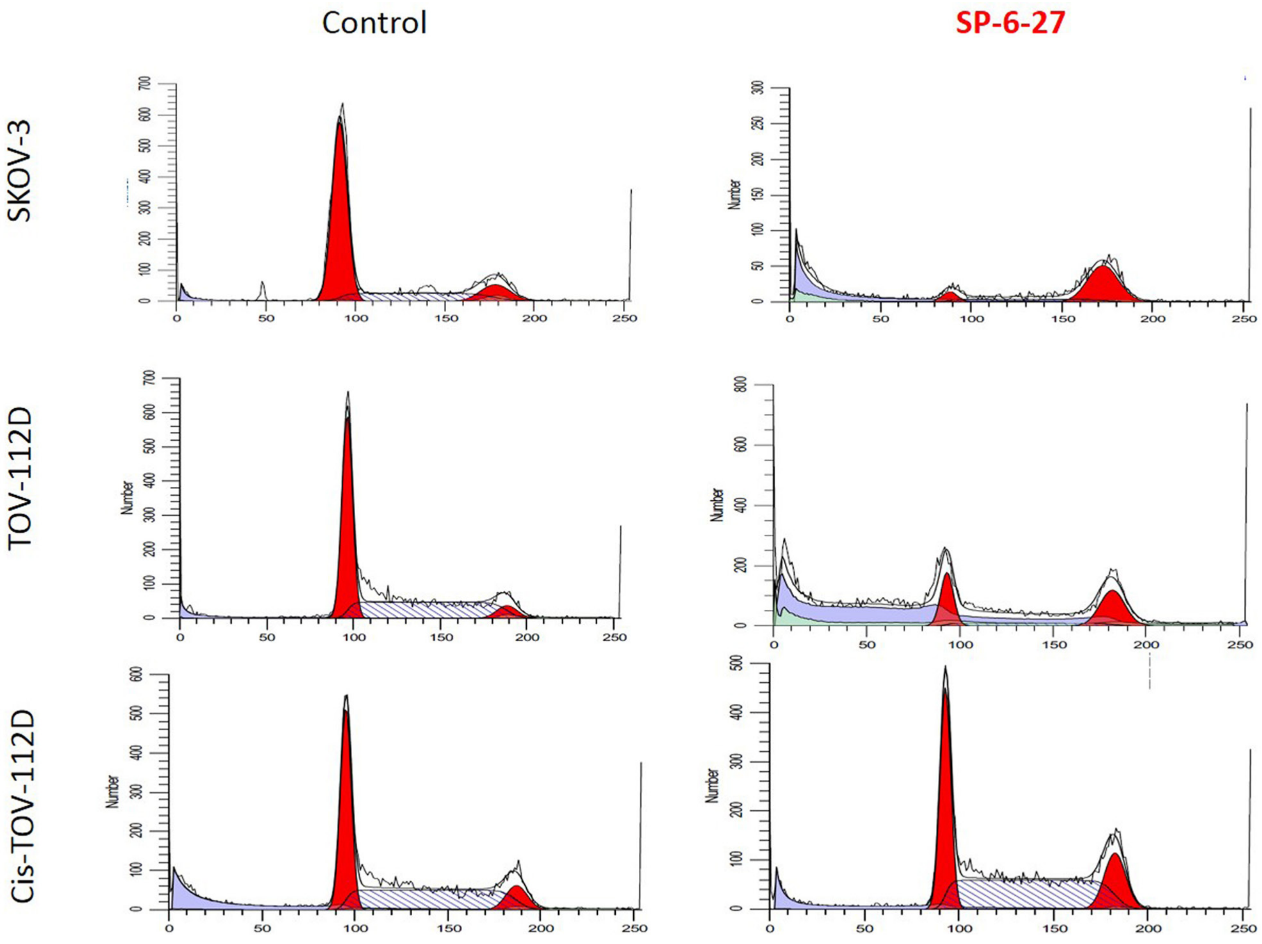

Supplementary Figure 3: Ovarian cancer cells arrest in G2-M phase upon SP-6-27 treatment.

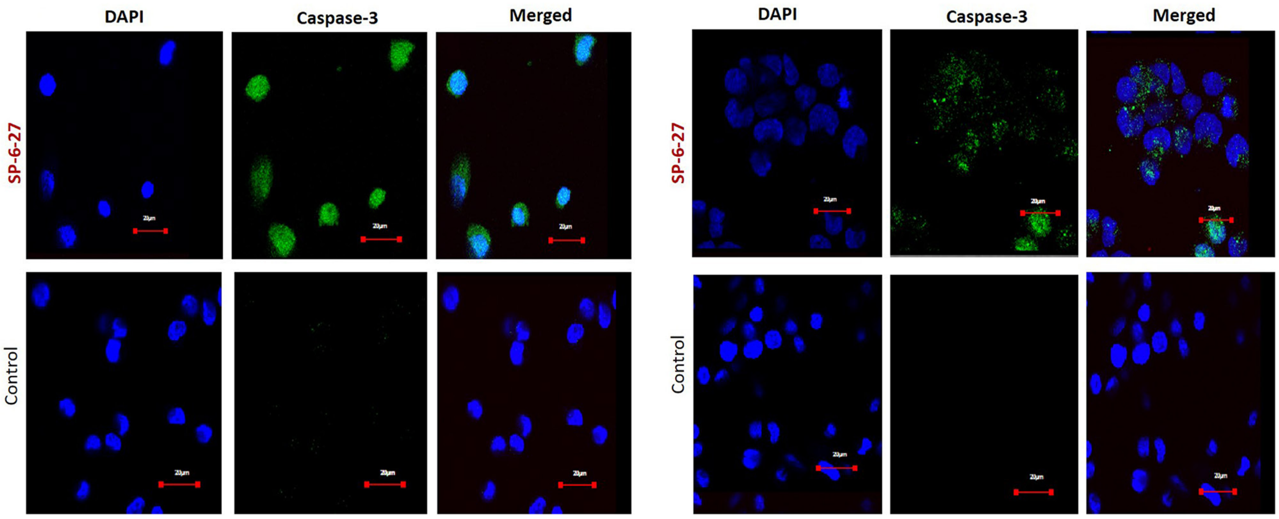

Supplementary Figure 4: Enhanced expression of cleaved-active caspase-3 in ovarian cancer cells following SP-6-27 treatment as determined by confocal microscopy analysis.

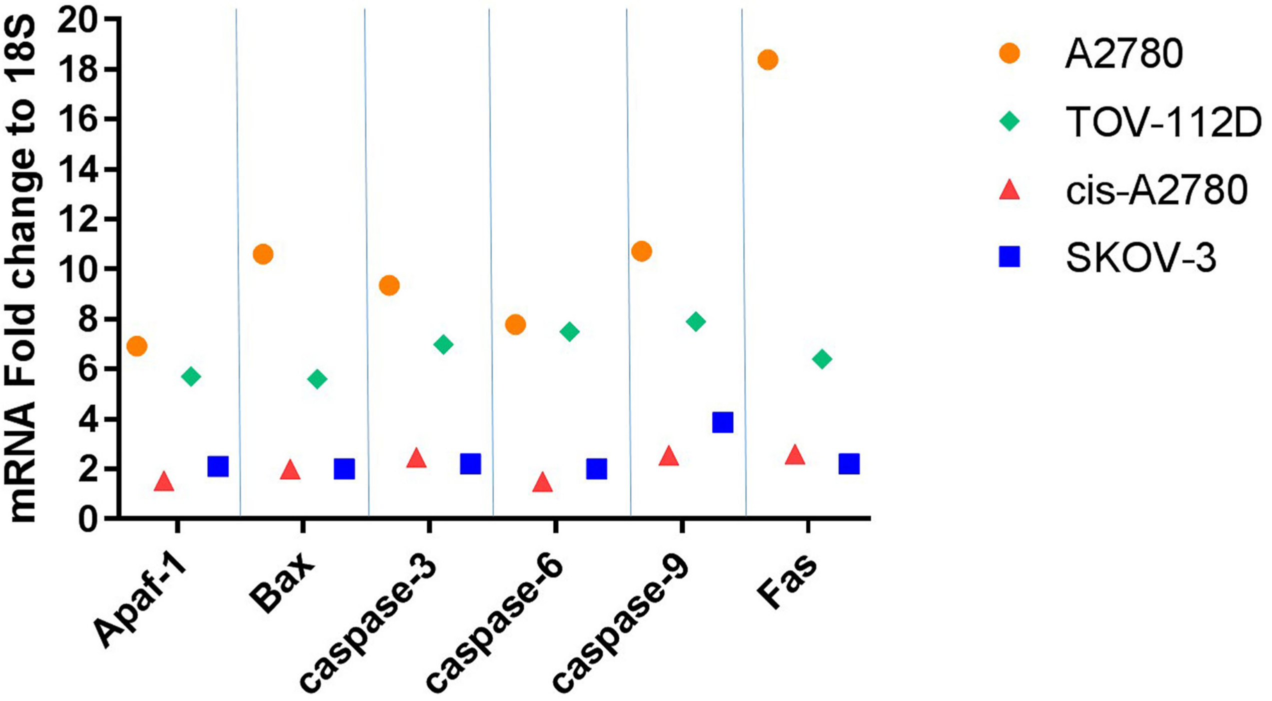

Supplementary Figure 5: Q-RT-PCR validation of upregulated cell death pathway array genes in different ovarian cancer cell lines.
